# Planning strategies for robust carbon-ion scanning radiotherapy for stage I esophageal cancer: a retrospective study

**DOI:** 10.1093/jrr/rrad057

**Published:** 2023-08-23

**Authors:** Makito Suga, Yohsuke Kusano, Yosuke Takakusagi, Yukio Oosawa, Shinichi Minohara, Daisaku Yoshida, Hiroyuki Katoh, Tadashi Kamada, Masataka Komori

**Affiliations:** Radiological and Medical Laboratory Sciences, Nagoya University Graduate School of Medicine, 1-1-20 Daiko-Minami, Higashi-ku, Nagoya City, Aichi, 461-8673, Japan; Section of Radiation Therapy Technology, Kanagawa Cancer Center, 2-3-2 Nakao, Asahi-ku, Yokohama City, Kanagawa, 241-8515, Japan; Section of Medical Physics and Engineering, Kanagawa Cancer Center, 2-3-2 Nakao, Asahi-ku, Yokohama City, Kanagawa, 241-8515, Japan; Department of Radiation Oncology, Kanagawa Cancer Center, 2-3-2 Nakao, Asahi-ku, Yokohama City, Kanagawa, 241-8515, Japan; Section of Radiation Therapy Technology, Kanagawa Cancer Center, 2-3-2 Nakao, Asahi-ku, Yokohama City, Kanagawa, 241-8515, Japan; Section of Medical Physics and Engineering, Kanagawa Cancer Center, 2-3-2 Nakao, Asahi-ku, Yokohama City, Kanagawa, 241-8515, Japan; Department of Radiation Oncology, Kanagawa Cancer Center, 2-3-2 Nakao, Asahi-ku, Yokohama City, Kanagawa, 241-8515, Japan; Department of Radiation Oncology, Kanagawa Cancer Center, 2-3-2 Nakao, Asahi-ku, Yokohama City, Kanagawa, 241-8515, Japan; Department of Radiation Oncology, Kanagawa Cancer Center, 2-3-2 Nakao, Asahi-ku, Yokohama City, Kanagawa, 241-8515, Japan; Radiological and Medical Laboratory Sciences, Nagoya University Graduate School of Medicine, 1-1-20 Daiko-Minami, Higashi-ku, Nagoya City, Aichi, 461-8673, Japan

**Keywords:** carbon-ion scanning radiotherapy, esophageal cancer, PTV margin, inter/intrafractional motion, robust planning, CT value replacement

## Abstract

This study aimed to establish a treatment planning strategy with carbon-ion scanning radiotherapy (CIRTs) for stage I esophageal cancer. The clinical data of seven patients treated with CIRTs were used. The setup error and interfractional and intrafractional motion error were analyzed using in-room computed tomography (CT) images for each treatment day. Finally, the planning target volume (PTV) margin was identified according to the accuracy of the treatment system. To ensure robustness against the positional displacements of the target and organs at risk (OAR), the replacement areas were placed as a contour adjacent to the tumor or OAR on the CT-image. The CT values of these areas were replaced by those of the target or OAR. Further, the dose distributions were optimized. Moreover, the variations in the target coverage from the initial plan for each treatment day (ΔV95%) were evaluated. By contrast, the risk of OAR was not evaluated in this study. The setup error was within 1.0 mm. The interfractional and intrafractional target motion errors were 2.8 and 5.0 mm, respectively. The PTV margins were 6.5 and 6.8 mm in the axial and depth directions, respectively. The robustness to target and OAR displacement was evaluated. The results showed that the target coverage with replacement could suppress decreased target coverage more than that without replacement. The PTV determination and replacement methods used in this study improved the target coverage in CIRTs for stage I esophageal cancer. Despite the need for a clinical follow-up, this method may help to improve clinical outcomes.

## INTRODUCTION

Esophageal cancer is the sixth most common cause of death due to malignancy worldwide, accounting for 5.3% of all cancer-related deaths [1]. Radiotherapy is one of the curative treatments for esophageal cancer, and good results have been reported for stage I esophageal cancer with this treatment modality [2]. In contrast, high doses to organs at risk (OAR), such as the lungs and heart, are detrimental to the patient’s long-term survival [3–5].

Several studies on esophageal cancer have shown that particle therapy is superior to intensity-modulated radiotherapy for dose reduction to OAR. However, most reports are related to proton beam therapy [6–8]. Carbon-ion radiotherapy (CIRT), which has superior physical/biological advantages [9, 10], is a promising treatment option for esophageal cancer. A previous study showed that CIRT and conventional photon-beam therapy have a favorable dose distribution [11]. In addition, preoperative CIRT for esophageal cancer has good therapeutic efficacy [12]. The carbon-ion scanning radiotherapy (CIRTs) system (CI-1000, TOSHIBA Corporation, Tokyo, Japan) was installed at Kanagawa Cancer Center (KCC), and the clinical trial was initiated in 2015 [13]. In all cases at our facility, CIRTs was administered using the raster scanning method, which involves scanning with a pencil beam with a diameter of ~3 mm at 1-sigma at the isocenter. This method, coupled at high speed, irradiates the target in a 3D manner and enables radiation dose distribution that provides greater concentrations compared with the conventional broad beam method [14]. For moving target irradiation, efforts have been made to establish a uniform irradiation field. It is achieved by controlling the movement of the target within 5 mm in the irradiation area, six rescanning per slice and completing the irradiation of one slice within two respiratory cycles [15]. However, these promising results and techniques could be compromised by different uncertainties associated with particle therapy and changes in beam range because of density variations [16].

In general, the assignment of a margin is based on uncertainty [17]. Margins are granted to geometrically magnify the target. In particle therapy, beam direction-specific margins must be set to account for the various uncertainties associated with actual dose delivery, such as patient setup accuracy, organ position variation and accuracy of range calculations [18]. Recently, robust optimization [19] has been proposed to cover the clinical target volume (CTV) by prescribed dose and to consider specific scenarios (range and setup uncertainties). This is considered in the case of multiple field optimization (MFO), in which the target dose is achieved in the target by simultaneously optimizing the spot weights of multiple ports. However, MFO is outside the scope of this study.

The accurate delivery of the target dose to the tumor is important to improve outcomes. In addition, the dose delivered to normal organs must be suppressed to decrease the risk of organ damage. In this study, we determined the PTV margin in CIRTs for stage I esophageal cancer. Furthermore, the robust planning method for addressing the intrafractional and interfractional motions of the tumor or OARs based on CT value replacement was examined.

## MATERIALS AND METHODS

### Patients

We included consecutive patients who underwent CIRTs for esophageal cancer at KCC from September 2020 to April 2023. The eligibility criteria for CIRTs were as follows: (i) patients who were histopathologically diagnosed with squamous cell carcinoma (SCC) or adenocarcinoma, (ii) those diagnosed with cT1bN0M0 according to the UICC 7th edition and (iii) those without prior treatment for esophageal cancer.

The characteristics of the patients included in the study are shown in Table 1. The study participants comprised five men and two woman, with a median age of 69.4 years (range, 59–82 years). The histopathological type was SCC in all patients. Tumor localizations were cervical esophagus, upper thoracic esophagus and mid-thoracic esophagus in one, one and three patients, respectively.

**Table 1:** Patient characteristics

Patient no.	Age	Gender	PS	Histology	Location (number of clips)	Number of in-room CT scans
1	77	Male	0	SCC	Ce (1)	4
2	59	Male	0	SCC	Mt (2)	4
3	66	Male	0	SCC	Ut/Mt (2)	4
4	82	Female	0	SCC	Ut (2)	3
5	59	Male	0	SCC	Ut (2)	3
6	73	Male	0	SCC	Ut (0)	3
7	70	Female	0	SCC	Ut (2)	3

Abbreviations: No., number; PS, performance status; SCC, squamous cell carcinoma; Ce, cervical esophagus; Mt, middle esophagus; Ut, upper thoracic esophagus.

### Contouring

Patients were placed in supine and prone positions on a vacuum mattress (BlueBAG: ElektaAB, Stockholm, Sweden) and secured with thermoplastic shells (Shellfitter: Kuraray, Tokyo, Japan). For all patients, computed tomography (CT) images of the maximum inhalation timing (In-peak) and the maximum exhalation timing (Ex-peak) were acquired using a treatment planning CT scanner (Aquilion LB, Canon Medical Systems Corporation, Tochigi, Japan) under free breathing.

**Fig. 1:**
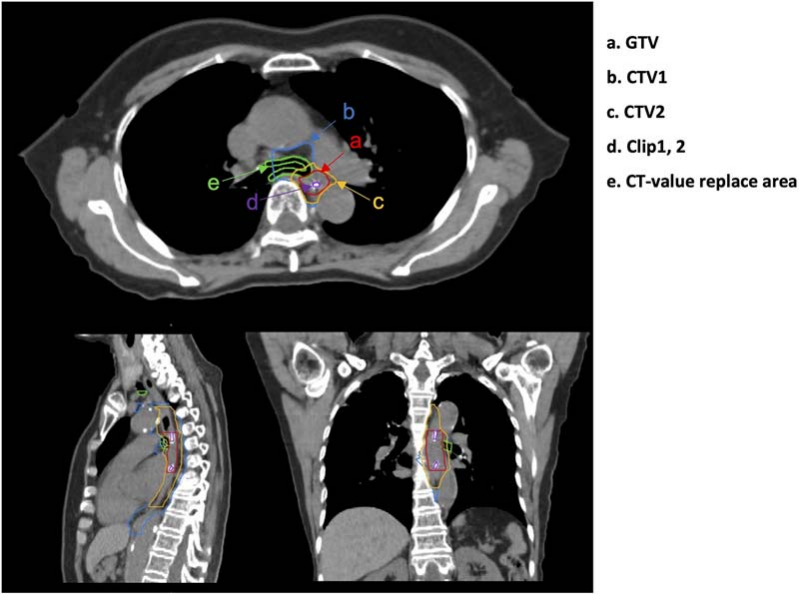
Axial, sagittal and coronal views of the structures for stage I esophageal cancer. GTV, gross tumor volume; CTV, clinical target volume.

MIM maestro version 6.9 (MIM Software Inc., Cleveland, OH, USA) was used for contouring. Gross tumor volume (GTV) was identified using an endoscopically implanted clip before obtaining initial planning CT scan (Fig. 1a). Clips were classified as clips 1 and 2 from the cephalic side; CTV1 included lymph node areas depending on the location of the primary tumor (Fig. 1b). The cervical esophagus included cervical, supraclavicular and superior mediastinal lymph nodes; the upper thoracic esophagus included supraclavicular and superior mediastinal lymph node areas; the middle thoracic esophagus included upper and lower mediastinal and intraperitoneal lymph node areas; the lower thoracic esophagus included lower mediastinal and intraperitoneal lymph node areas. Owing to the size of the irradiation field (20 × 20 cm) [13], it might not have been possible to include the complete lymph node area in the cephalocaudal direction. Therefore, CTV1 was set to a maximum of 20 cm from the sternal notch in the cephalocaudal direction in such cases. CTV2 was enlarged by 3 cm along the long axis of the esophagus from the GTV and 5 mm in the lateral and dorsoventral directions (Fig. 1c). GTV, CTV1 and CTV2 were drawn on In-peak and Ex-peak CT images, and their contours were combined to create internal GTV (IGTV), internal CTV 1 (ICTV1) and ICTV2. In addition to the target drawings, the lungs, heart and spinal cord were drawn on Ex-peak CT images used for optimization calculations.

### Planning target volume margin

The planning target volume (PTV) margin was determined per ICRU Report 62 [17]. The setup margin (SM) and internal margin (IM) were derived. SM was derived separately for the direction perpendicular to the beam axis (plane) and for the direction along the beam axis (depth) [20]. Organ position variation during irradiation was excluded from IM because it is considered in IGTV and ICTV. Subsequently, the total margin (TM) was calculated for each direction based on the square root of the sum of squares.


(1) \begin{equation*} {\mathrm{TM}}_{\mathrm{Plane}}=\sqrt{{{\mathrm{SM}}_{\mathrm{Plane}}}^2+{\mathrm{IM}}^2} \end{equation*}

(2) \begin{equation*} {\mathrm{TM}}_{\mathrm{Depth}}=\sqrt{{{\mathrm{SM}}_{\mathrm{Depth}}}^2+{\mathrm{IM}}^2} \end{equation*}

PTV1 and PTV2 were created by adding margins to ICTV1 and ICTV2, respectively, based on TM values.

#### Setup error

For patient matching during treatment, we employed a 2D–3D bone matching system [21] using frontal- and lateral-view X-ray (2D) and Ex-peak CT images (3D). The patient positioning error was determined from the positional error between the digitally reconstructed frontal- and lateral-view X-rays created from the initial planning CT images and the final patient setup X-rays [22].

The position error was divided into target registration error (TRE) and angular error (AE). *k* represents the number of treatments.


(3) \begin{equation*} {\mathrm{TRE}}_k=\sqrt{\Delta{x_k}^2+\Delta{y_k}^2+\Delta{z_k}^2} \end{equation*}

(4) \begin{equation*} {\mathrm{AE}}_k=\sqrt{\Delta{\psi_k}^2+\Delta{\phi_k}^2+\Delta{\theta_k}^2} \end{equation*}

#### Intrafractional target motion error

The intrafractional target motion was evaluated in the left–right (LR), superior–inferior (SI) and anterior–posterior (AP) directions using X-ray serial images acquired during the initial treatment planning to evaluate the amount of displacement of the clip over time given the maximum exhalation. X-ray serial imaging, a type of cine imaging performed using a dynamic flat-panel detector (DFPD; DAR8000f, Shimadzu Cop. Kyoto, Japan), was performed immediately before obtaining the initial planning CT scan image. In DFPD imaging, the following parameters were used: an area of 43 × 43 cm, a pixel pitch of 0.15 mm, and an amorphous selenium (aSe) photoconductor with a maximum of 30 frames per second [23]. The distances from the room isocenter to FPD and from the X-ray source to FPD were 155 and 213 cm, respectively. Changes in the LR and SI directions over time were acquired from front-view X-ray serial images, whereas changes in the AP and SI directions were acquired from lateral-view X-ray serial images. To measure respiratory variability over time, five X-ray serial imaging sessions were performed per patient per examination. The 3D displacement of the clip given maximum exhalation, acquired in each imaging session, was evaluated.

#### Interfractional target motion error

Interfractional target motion was evaluated using initial planning and in-room CT images to evaluate the 3D displacement of the GTV with exhalation during the treatment period based on the isocenter position. In-room CT scans were acquired at least once a week during the treatment period, considering each patient’s physical condition and X-ray exposure. Ex-peak CT images were acquired while maintaining the patient setup at the time of treatment. The in-room CT scanner used was the same as the treatment planning CT scanner. GTV was contoured on the in-room CT image. The center of gravity of the GTV on Ex-peak CT image of the initial planning and the GTV on the in-room CT image were analyzed to evaluate the 3D displacement.

## INITIAL PLANNING

Monaco version 5.20 for carbon (Elekta AB, Stockholm, Sweden) was used for the initial planning. The total dose was 50.4 GyRBE/12 fractions: 33.6 GyRBE/8 fractions for PTV1 and 16.8 GyRBE/4 fractions for PTV2. The gantry angles for beam injection were 0° and 180*°*, and the beam was equally irradiated six times from each angle. The scanning method was selected as the irradiation method. A CIRT treatment plan was established to cover 95% of the PTV with 95% of the prescription dose (V95%). If normal organs were adjacent to the PTV, the dose was reduced based on adherence to dose constraints for normal organs. The percentage of the lung volume receiving at least 20 GyRBE (V20) was <10% for the lungs, the mean dose (*D*_mean_) for the heart was <20 GyRBE [24], and the maximum dose (D_max_), which covered 0.01 cm^3^ of the spinal cord, was 30 GyRBE [25].

### Calculation of the treatment dose distributions using the in-room computed tomography images

Ex-peak CT images were acquired using an in-room CT scanner while maintaining the patient setup at the time of treatment. GTV, CTV1, CTV2 and OAR were drawn on the in-room CT images and transferred to the treatment planning device. The isocenter position at the time of treatment was set based on the markers projected on the in-room CT image. Subsequently, without changing the irradiation conditions (spot position, number of particles in each spot, etc.) set in the initial planning, the dose distribution during the treatment was calculated based on the isocenter position for each treatment day.

### Analysis of the target coverage

The variations in target coverage (ΔTC) in V95% from the initial plan were defined using the following formula:


(5) \begin{equation*} \Delta \mathrm{TC}={\mathrm{TC}}_{\mathrm{IRCT}}-{\mathrm{TC}}_{\mathrm{Plan}}, \end{equation*}

where TC_IRCT_ and TC_Plan_ are the target coverages based on fractional and initial dose distributions, respectively. The ΔTC was evaluated for CTV1 and CTV2.

### Statistical analysis

In the current study, the evaluation was performed using serial X-ray and in-room CT scan images. During the assessment of intrafractional target motion for each direction (LR, AP and SI) using serial X-ray images and interfractional target motion for each direction using in-room CT images, no normality was observed in each data set. Therefore, the Friedman test was used as a three-group evaluation of quantitative data. Given that the three groups could be evaluated three times, the *P* values obtained were multiplied by three using the Bonferroni method. Furthermore, when assessing ∆TC changes with and without replacement using in-room CT scan images, no normality was observed in each data set. The Wilcoxon signed-rank test was used as a two-group evaluation of quantitative data. A *P*-value of <0.05 were considered statistically significant. Statistical analyses were performed using the Statistical Package for the Social Sciences software (version 26.0, IBM Inc., Armonk, NY, the USA).

## RESULTS

### Setup Error

The mean setup errors were TRE = 0.4 mm and AE = 0.2° (Table 2). Considering the positioning accuracy of the 6-axis robotic arm (0.5 mm) and the calculation accuracy of the 2D–3D bone matching software (0.3 mm, 0.3°) [22] and compared with a previous study on the trunk (TRE = 1.1 ± 1.2 mm, AE = 0.6 ± 0.4°) [26], our study showed a good setup accuracy. Based on this finding, the setup error was set to 1.0 mm.

**Table 2:** Patient setup error

Patient no.	TRE (mm)	AE (deg)
	Mean ± SD (Max–Min)	Mean ± SD (Max–Min)
1	0.6 ± 0.3 (1.3–0.3)	0.2 ± 0.1 (0.5–0.1)
2	0.5 ± 0.4 (1.1–0.1)	0.1 ± 0.1 (0.3–0.0)
3	0.4 ± 0.2 (0.7–0.0)	0.2 ± 0.2 (0.6–0.0)
4	0.5 ± 0.2 (0.9–0.4)	0.2 ± 0.2 (0.5–0.0)
5	0.3 ± 0.1 (0.4–0.1)	0.0 ± 0.0 (0.1–0.0)
6	0.3 ± 0.1 (0.5–0.1)	0.1 ± 0.1 (0.2–0.0)
7	0.2 ± 0.2 (0.4–0.0)	0.1 ± 0.1 (0.3–0.0)
Total	0.4 ± 0.4 (1.3–0.0)	0.2 ± 0.2 (0.6–0.0)

Abbreviations: No., number; TRE, target registration error; AE, angular error; SD, standard deviation; Max, maximum; Min, minimum.

### Intrafractional target motion error

**Fig. 2:**
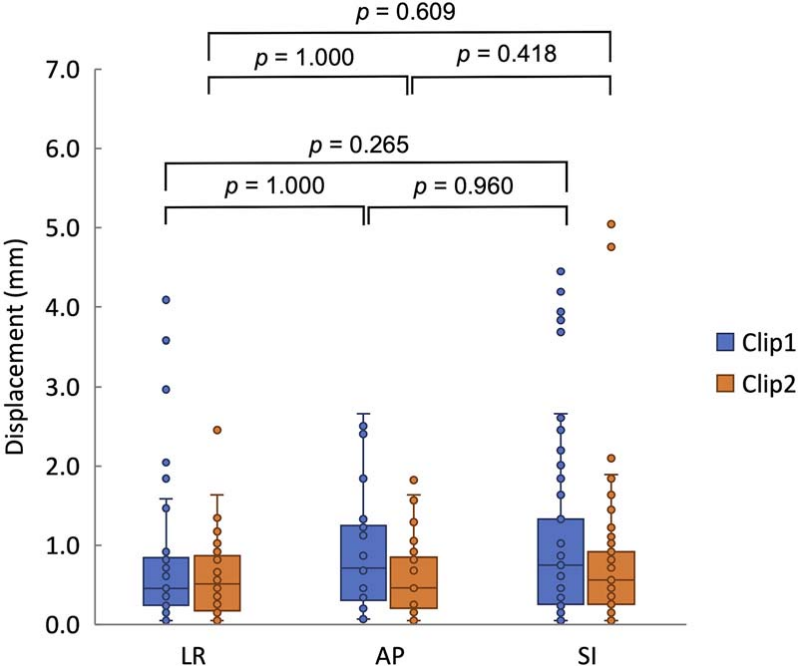
Intrafractional target motion deviation between different directions. For each direction, the median, first quartile, third quartile, maximum and minimum values and outliers for displacement are presented in a box-and-whisker plot. Statistical analyses were then performed using the Friedman test. LR: left–right; AP: anterior–posterior; SI: superior–inferior.

At our institution, initial planning was performed so that the beam was irradiated at a time when the respiratory displacement of the tumor was <5.0 mm [27]. The measured positional displacement of the clip at maximum exhalation was ~5.0 mm, and the maximum displacement, excluding outliers, in each axis was ~2.8 mm (Fig. 2). Clip 1 on the proximal side had a slightly larger displacement in the LR direction. This may be attributed to the influence of the heartbeat. Clip 2 on the distal side exhibited a slightly larger movement in the cephalocaudal direction. This may have been caused by the effects of respiratory migration. In each direction (LR, AP and SI), the amount of movement of clips 1 and 2 did not differ significantly. Therefore, the intrafractional target motion error was set at 2.8 mm.

### Interfractional target motion error

**Fig. 3:**
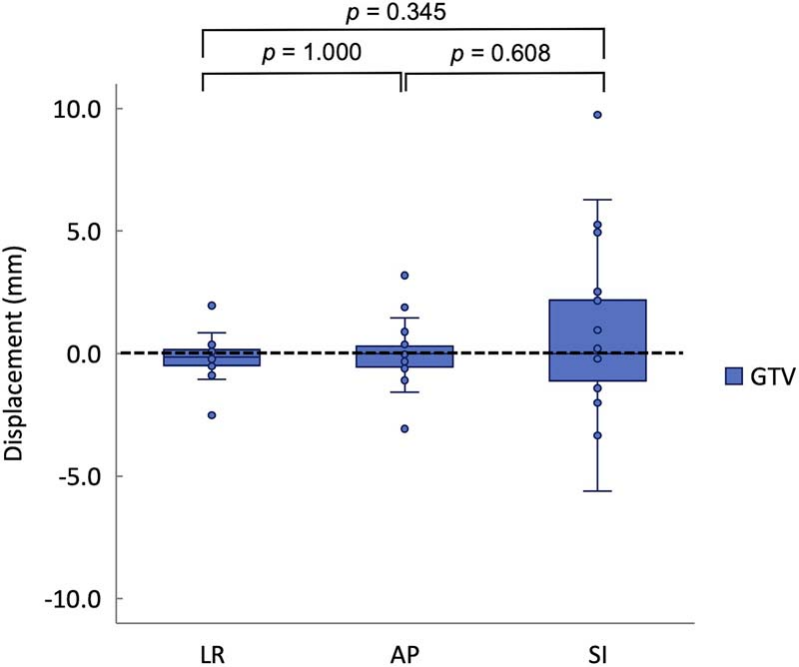
Interfractional target motion deviation between different directions. For each direction, the median, first quartile, third quartile, maximum and minimum values and outliers for displacement are presented in a box-and-whisker plot. Statistical analyses were then performed using the Friedman test. GTV, gross tumor volume; LR: left–right; AP: anterior–posterior; SI: superior–inferior.

Interfractional target motion was larger in the SI direction than in the other directions, with a maximum value of ~5.0 mm observed when outliers were excluded (Fig. 3). There was no significant difference between each direction. Therefore, the interfractional target motion error was set at 5.0 mm.

### Planning target volume margin

The allowable values of the irradiation device and the SM and IM derived in this study are shown in Table 3. The tolerance of the irradiation device is the threshold value to stop the beam, and the actual performance value is smaller than the tolerance. The PTV margin was adopted as an acceptable value because it is the margin related to therapeutic irradiation. The SM was 3.2 mm in the axial direction and 3.7 mm in the depth direction. The IM was 5.7 mm based on the square root of the sum of the squares of the inter/intrafractional target motions. Consequently, the PTV margin (i.e. the TM) calculated from the square root of the sum of the squares of SM and IM in each direction was 6.5 mm in the body axis direction and 6.8 mm in the depth direction.

**Table 3:** Accuracy of the system used to determine setup margins for perpendicular-to-beam and beam-axis directions

Setup margin plane (SM_Plane_)		Value (mm)	Internal margin (IM)		Value (mm)
a	Beam axis	0.5	m	Respiratory motion (included in IGTV)	0.0
b	Spot position (X)	2.0
c	Spot position (Y)	2.0	n	Intra-factional motion	2.8
d	Isocenter	0.5	o	Inter-factional motion	5.0
e	X-ray tube	0.5	$\sqrt{m^2+{n}^2+{o}^2}$		**5.7**
f	FPD	0.5			
g	Patient setup	1.0			
$\sqrt{a^2+{b}^2+{c}^2+{d}^2+{e}^2+{f}^2+{g}^2}$		**3.2**			
**Setup margin depth (SM_ Depth_)**		**Value (mm)**	**Total margin (TM)**		**Value (mm)**
h	CT-SP table	3.0	${\mathrm{TM}}_{\mathrm{Plane}}=\sqrt{{{\mathrm{SM}}_{\mathrm{Plane}}}^2+{\mathrm{IM}}^2}$		**6.5**
i	Range	1.0	${\mathrm{TM}}_{\mathrm{Depth}}=\sqrt{{{\mathrm{SM}}_{\mathrm{Depth}}}^2+{\mathrm{IM}}^2}$		**6.8**
j	Spot position (Z)	2.0			
$\sqrt{h^2+{i}^2+{j}^2}$		**3.7**			

Values in bold mean calculated margin values. Abbreviations: SM, setup margin; IM, internal margin; TM, total margin; FPD, flat-panel detector; CT-SP, computed tomography value-stopping power ratio; IGTV, internal gross tumor volume.

The value related to the treatment beam indicates the allowable value of interlock occurrence.

### Variation of the target coverage

**Fig. 4:**
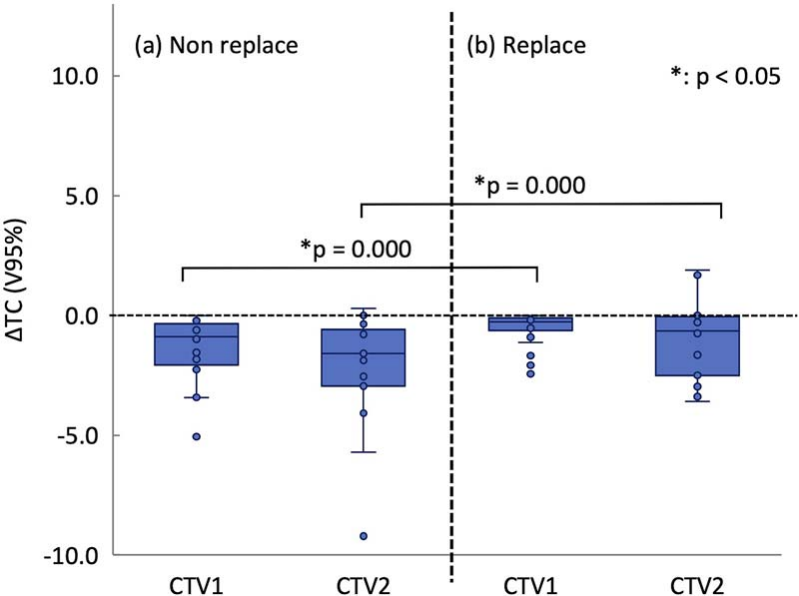
Evaluation results for variations in CTV1, 2 coverage (ΔTC). ΔTC values with (**a**) Not replacement and (**b**) replacement. For each method, the median, first quartile, third quartile, maximum and minimum values and outliers for ΔTC are presented in a box-and-whisker plot. Statistical analyses were then performed using the Wilcoxon signed-rank test. The asterisk (*) indicates that there is a significant difference between them. CTV, clinical target volume.

Figure 4a shows the box-and-whisker plots of variation of the ΔTC from the initial plan. The ΔTC was more likely to decrease for CTV1 and CTV2 compared with the initial plan. The decrease in target coverage from the initial plan for CTV2 was greater than that for CTV1, with median values of −0.88% and −1.59%, respectively. The factors that may have contributed to lower target coverage were changes in patient physique and changes in beam range due to displacements of OAR position near the target.

## DISCUSSION

In this study, the appropriate PTV margins for CIRTs for stage I esophageal cancer were calculated considering the accuracy of the treatment system.

The movement of the esophagus under free respiration is considered small because it is restricted by the surrounding organs [28]. Regarding intrafractional target motion, Jin *et al*. [29] used 4DCT to analyze the intrafractional target motion of 60 markers inserted in 20 patients with esophageal cancer under free respiration. The results revealed that the intrafractional target motion (LR/AP/CC direction) was 1.5/1.6/2.9 mm for the proximal esophagus, 1.5/1.4/3.7 mm for the middle esophagus and 2.6/3.3/5.4 mm for the distal esophagus. Lever *et al*. [30] measured esophageal cancer movement via cine magnetic resonance imaging and found that tumor movement was greater in the SI direction than in the AP or LR direction. These results are consistent with those of the present study and may be attributable to the effect of respiration [31]. Regarding interfractional target motion, Wang *et al*. [28] in their study on thoracic esophageal cancer found no significant difference in the amount of movement by direction. However, Wang *et al*. [32] found that interfractional target motion in esophageal cancer was greater in the SI direction than in other directions, which is similar to the findings of the present study.

Our results showed that the dose distribution recalculated by in-room CT showed a decrease in target coverage (Fig. 4a). Irie *et al*. [33] found that changes in tumor displacement and water equivalent thickness affect the robustness of the dose distribution. In this study, the possible causes of reduced target coverage include changes in patient physique and changes in beam range due to the displacement of the OAR position near the target. Target position displacement is considered in the PTV margin as IM. To improve target coverage and accurately deliver the dose to the tumor, it is necessary to develop a treatment plan that is robust to these changes.

First, the influence of changes in the patient’s body shape during the treatment period on target coverage was examined. The body shape difference between the initial plan and in-room CT images was prepared as a contour, and the body shape in the in-room CT image was virtually matched to that in the plan CT image by rewriting the CT values ​​of the contour with those of fat or air. The dose distribution was calculated on in-room CT images after body shape adjustment without changing the irradiation conditions determined in the initial planning, and the change in target coverage from the initial plan was calculated to evaluate the effect of changes in body size [16]. Our results revealed that the effect of body size change was −0.2% for CTV1 and −0.6% for CTV2, indicating that the effect of changes in patient body thickness during the treatment period on target coverage was minimal.

**Fig. 5:**
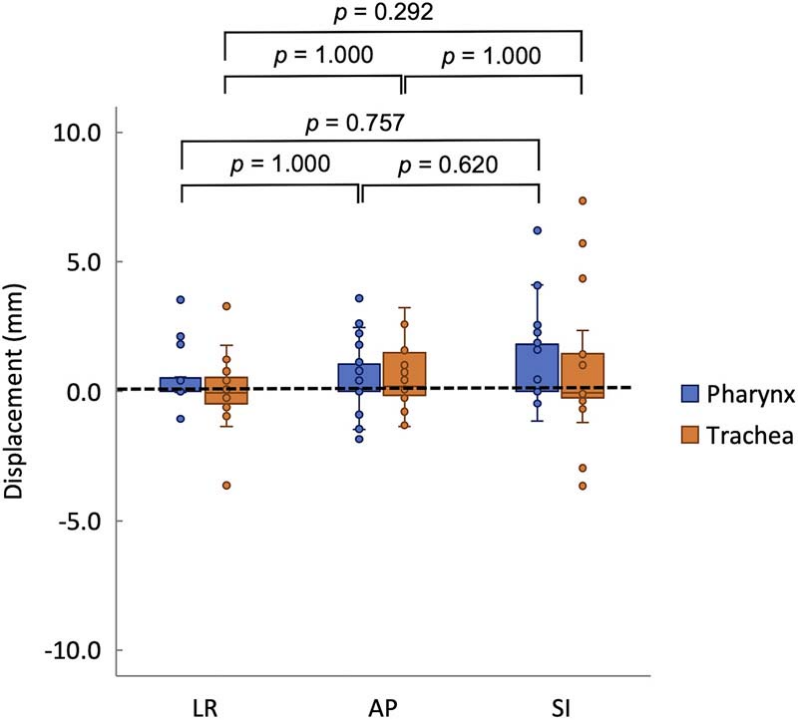
Interfractional OAR motion deviation between different directions. For each direction, the median, first quartile, third quartile, maximum and minimum values and outliers for displacement are presented in a box-and-whisker plot. Statistical analyses were then performed using the Friedman test. LR: left–right; AP: anterior–posterior; SI: superior–inferior.

Next, the effect of change in the OAR position variation was additionally examined. The 3D positional displacement of the OAR during the treatment period (i.e. interfractional OAR position variation) was evaluated. The analysis was performed on the pharynx and trachea. These are air masses with a constant volume, and the density change due to their position displacement was considered to have a significant effect on the dose distribution. The results are shown in Fig. 5. The OAR was slightly larger in the SI direction but not significantly different in each direction. Because the carbon-ion beam range is highly sensitive to density changes, sparse density changes along the beam path directly lead to changes in the dose distribution. Botas *et al*. [34] demonstrated that during proton intensity-modulated therapy for lung cancer, density replacement within the IGTV enables treatment planning that ensures robustness against interfractional motion of the target. Kusano *et al*. [16] also demonstrated that during CIRTs of pancreatic cancer, the CT value of the gastrointestinal tract contour can be rewritten by the average CT values that include gastrointestinal tract gases to ensure a treatment plan that is robust against gastrointestinal tract gases.

Therefore, based on the abovementioned studies, we investigated the rewriting of the air layer near the target and OAR with the CT value of the target or OAR (Fig. 1e) to assess the robustness to position changes of the target and OAR. The dose distribution was optimally calculated using the initial plan CT scan images using the replacement process, and the initial plan was created under the replacement condition. Thereafter, the dose distribution was calculated using in-room CT images without changing the irradiation conditions of the initial plan. Finally, the change in target coverage when the air layer was rewritten with the CT value of the target or OAR was determined (Fig. 4b). Results revealed that the ΔV95% of the treatment plan optimized with the CT value replacement was significantly better than that optimized without the CT value replacement (Fig. 4a). In addition, the OAR dose was within the clinically acceptable range in all cases.

This study showed that PTV margin with consideration of the various types of errors and the CT value replacement method for interfractional target motion and OAR position variation can be used to develop a robust treatment plan for CIRTs for esophageal cancer. Furthermore, the robustness of treatment planning could be further improved by selecting the optimal gantry angle. The esophageal cancer motion under free breathing is small. However, the intrafractional dose change is significant [35] owing to differences in gantry angle. This finding could be ascribed to the effects of the heart and different gastric gas fills. The most robust irradiation angles for esophageal cancer are the anterior (0°) and posterior (180°) angles [36]. This is because the diaphragm is attached to the lumbar spine via the left and right cruciate tendons, and the posterior fan-shaped region containing the esophagus is relatively stationary with respect to diaphragmatic movement [37]. Based on the positioning of the target and OAR, in some cases, cardiac effects can be prevented, and the spinal cord dose can be reduced by gantry angling the beams from the anterior and posterior directions.

However, the study had several limitations. The number of cases and in-room CT images used in the study was small. Increasing the number of cases and in-room CT images may improve the accuracy of our findings. Nevertheless, the evaluation of dose distribution using in-room CT in CIRTs has rarely been reported; therefore, this study is of high clinical value. Furthermore, this study is considered valuable as it provides a treatment planning policy for carbon beam scanning treatment of esophageal cancer.

In conclusion, we have presented a method for determining the PTV margin and found that density replacement can be used to develop a robust treatment plan for patients undergoing CIRTs for esophageal cancer. This method does not require any specific software or equipment and can be implemented clinically in any facility. Implementing this method has the potential to improve target coverage and enhance clinical outcomes. However, the doses to OARs may increase owing to the fixed margin set for the replacement area. Furthermore, as shown in Fig. 4b, outliers were observed when using this method. Therefore, it is crucial to periodically monitor the patient’s body status through in-room CT scans. Although this study focused on esophageal cancer, it is important to recognize that anatomical changes vary based on the treatment site. For example, rectal gas poses challenges in prostate cancer, whereas tumor growth or fluid retention is observed in head and neck cancer, gastrointestinal gas in pancreatic cancer and tumor growth in bone and soft tissue sarcomas. Consequently, we believe that the procedures for preparing a robust treatment plan for anatomical changes differ depending on the treatment site.

## Data Availability

The original contributions presented in the study are included in the article. Further inquiries can be directed to the corresponding author.
